# Supplementary material for: Free-Radical-Mediated Formation of Trans-Cardiolipin Isomers, Analytical Approaches for Lipidomics and Consequences of the Structural Organization of Membranes
Source: Biomolecules. 2020 Aug 15;10(8):1189. doi: 10.3390/biom10081189 (PMC7465319; doi:10.3390/biom10081189)
Supplement: Supplementary file 1 [file biomolecules-10-01189-s001.pdf]

Supporting Material

Free Radical-Mediated Formation of Trans-Cardiolipin isomers, Analytical Approaches for Lipidomics and Consequences for the Structural Organization of Membranes

Fabrizio Vetica [1], Anna Sansone [1], Cesare Meliota [1], Gessica Batani [1], Marinella Roberti [2], Chryssostomos Chatgilialoglu [1,3,*] and Carla Ferreri [1,*]

[1] Istituto per la Sintesi Organica e la Fotoreattività, Consiglio Nazionale delle Ricerche, Via Piero Gobetti 101, 40129 Bologna, Italy; fabrizio.vetica@isof.cnr.it (F.V.); anna.sansone@isof.cnr.it (A.S.);. cesare.meliota@studio.unibo.it (C.M.); gessica.batani@studio.unibo.it (G.B.)

[2] Department of Pharmacy and Biotechnology, University of Bologna, Via Belmeloro 6, 40126 Bologna, Italy; marinella.roberti@unibo.it (M.R.)

[3] Center for Advanced Technologies, Adam Mickiewicz University, 61-614 Poznań, Poland

* Correspondence: chrys@isof.cnr.it (C.C.); carla.ferreri@isof.cnr.it (C.F.)

Optimization of the transesterification conditions

The procedure describes in the main text (section 2.1), was accurately determined in order to work under dry conditions. In fact, the inconsistency and not reproducibility of transesterification experiments of CL using HCL-MeOH and/or KOH/MEOH at different concentrations were caused probably by the presence of water (also in traces) formed during the reaction, slowing and negatively affecting the yield. The use of NaOMe sensibly reduced the problem of water-based hydrolysis, anyway a particular accuracy was required for the preparation of the NaOMe solution as described in the following procedure:

molecular sieves were activated by drying at 160° in the oven for one night and left to reach the room temperature under a stream of nitrogen; MeOH (HPLC grade) was added to the molecular sieves always under nitrogen and left in this condition for two hours. Afterwards, the weighted amount of metallic sodium to reach 22 mM required for the reaction was added with care to the calculated volume of MeOH, keeping all materials under nitrogen. Fresh reagent in solution was used each time.

Table S1 describes the conditions used and the results of the cardiolipin transesterification into fatty acid methyl esters (FAME), evidencing the formation of free fatty acids as products of the hydrolysis in the experimental conditions. Table 2 describes the results using the NaOMe protocol, avoiding formation of free fatty acids.

Table S1. Transesterification experiments on cardiolipin using KOH/MeOH.

Entry	KOH [M]	Time [min]	Yield [%] mean ± SD[a]	Hydrolysis[b]
1	0.5	10	50.4 ± 13.4	Yes
2	1	10	59.9 ± 5	Yes
3	2	10	21± 12.2	Yes

[a] Determined by GC calibration curves of the corresponding FAME peak areas. Yields are calculated considering that only 95% of the starting material contains 18:2 chains; [b] Determined by TLC

Table S2. Transesterification experiments on cardiolipin using NaOMe.

Entry	NaOMe [mM]	Time [min]	Yield [%] mean ± SD[a]	Hydrolysis[b]
1	11	330	51.8	Yes
2	11	1380	48.7	Yes
3	11	420	30.5	Yes
4	20	300	54.5	Yes
5	5	270	43	Yes
6[c]	22	120	25.6	Yes
7[d]	11	270	41.2 ± 15.1	Yes
8[d,e]	22	120	84.6 ± 2.6	No
9[d,e]	22	150	78.7 ± 2.9	No

[a] Determined by GC calibration curves of the corresponding FAME peak areas. Yields are calculated considering that only 95% of the starting material contain 18:2 chains; [b] Determined by TLC; [c], reaction mixture directly evaporated under vacuum, the residue dissolved in 1 mL of n-hexane and injected in GC; [d] Experiment performed in triplicate; [e] The reaction mixture was first treated with hexane (3×2 mL) containing 0.1 mL of a standard solution of 17:0 methyl ester, to extract FAMEs, than the combined organic phases were evaporated under *vacuum*, the residue dissolved in 1 mL of n-hexane and injected in GC (the GC areas were adjusted using a correction factor obtained by the recovery of the 17:0 standard).

The conditions used for cardiolipin transesterification into fatty acid methyl esters (FAME), were tested also on a representative phospholipid (1-palmitolyl-2-oleoyl-phosphatidyl choline, POPC) in order to evaluate the recovery yields and indicate the new transesterification conditions as the best method for biological samples.

Table S3. Transesterification experiments on POPC[a].

Reagent	Time [min]	Yield 16:0 [%] mean ± SD[b]	Yield 9*cis* 18:1 [%] mean ± SD[b]
KOH 0.5 M	10	96 ± 4.2	97 ± 1.4
NaOMe 22 mM	120	92 ± 2.8	94.5 ± 3.5

[a] Experiments performed in duplicates; [b] Determined by GC calibration curves of the corresponding FAME peak areas. Yields are calculated considering that only 95% of the starting material contains 18:2 chains.

NMR spectra

Bovine heart CL (starting material)

^{1}H NMR (500 MHz, Methanol-d4) δ 5.35 (tq, J = 10.9, 5.4, 3.9 Hz, 16H, 8x C*H*=C*H*), 5.23 (dt, J = 8.4, 4.2 Hz, 2H, 2x glycerol CH), 4.56 (bs, 3H, 3x OH), 4.46 (dd, J = 12.0, 3.1 Hz, 2H, glycerol CH_2), 4.20 (dd, J = 12.0, 6.7 Hz, 2H, glycerol CH_2), 4.01 (t, J = 5.4 Hz, 4H, glycerol CH_2), 3.98 – 3.86 (m, 4H, glycerol CH_2), 2.78 (t, J = 6.5 Hz, 8H, 4x CH=CHC*H*$_2$CH=CH), 2.33 (dt, J = 14.3, 7.4 Hz, 8H, 4x CH_2C*H*$_2$C(O)OR), 2.07 (q, J = 6.8 Hz, 16H, 4x C*H*$_2$CH=CHCH$_2$CH=CHC*H*$_2$), 1.61 (q, J = 6.9, 6.3 Hz, 8H, 4x C*H*$_2$CH$_2$C(O)OR), 1.41 – 1.27 (m, 56H, 4x14 CH_2), 0.91 (t, J = 6.9 Hz, 12H, 4x CH_3) ppm.

^{13}C NMR (126 MHz, Methanol-*d*$_4$) δ 174.85, 174.54, 130.96, 130.91, 130.90, 130.85, 129.11, 129.07, 72.01, 71.94, 67.77, 67.72, 64.83, 64.77, 64.73, 63.78, 35.16, 34.97, 32.68, 30.81, 30.78, 30.69, 30.49, 30.42, 30.38, 30.32, 30.29, 30.25, 30.23, 28.24, 28.22, 28.20, 26.60, 26.06, 26.03, 23.64, 14.46 ppm.

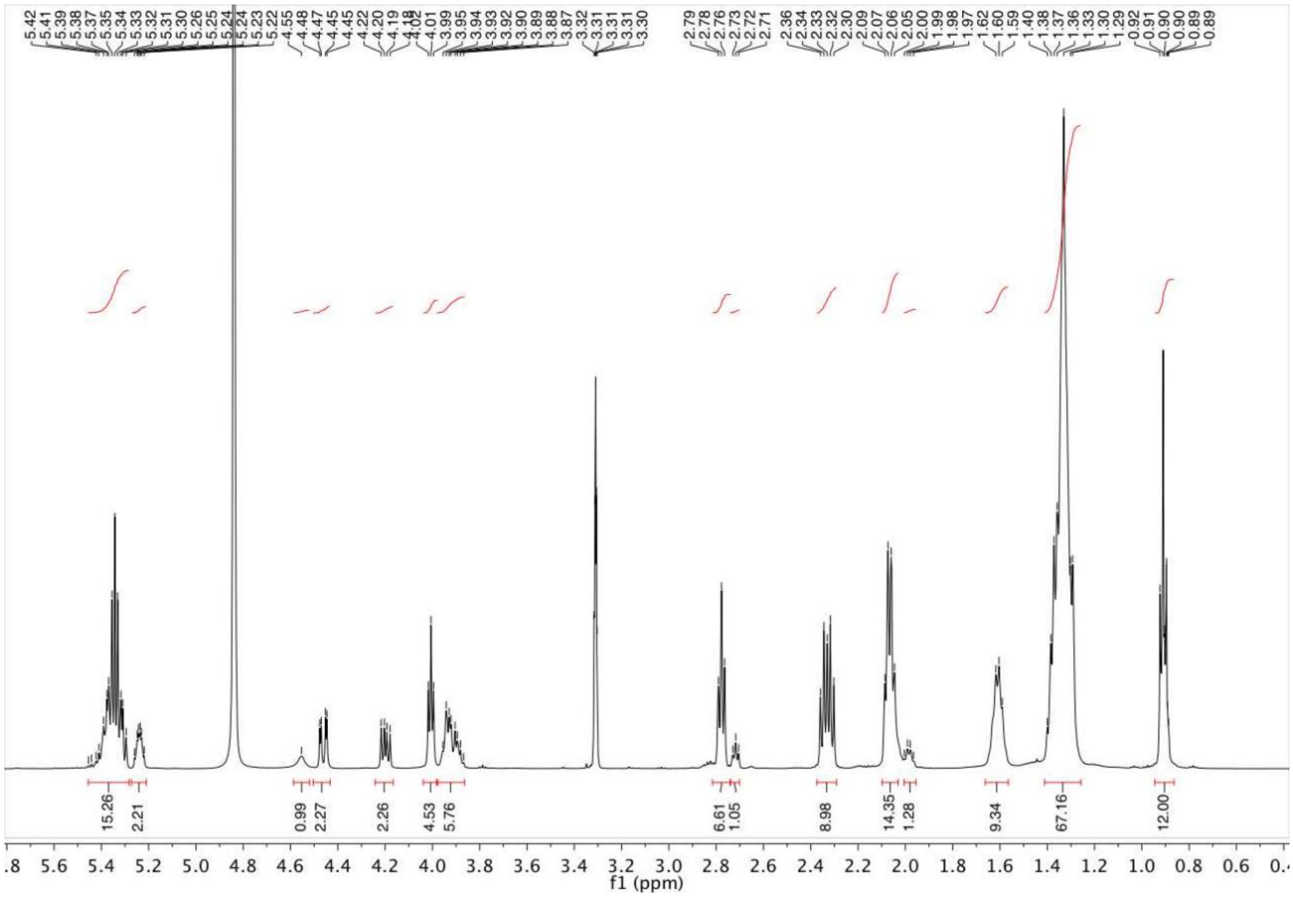

Figure S1. ^{1}H NMR spectrum of natural bovine hearth cardiolipin in CD_3OD

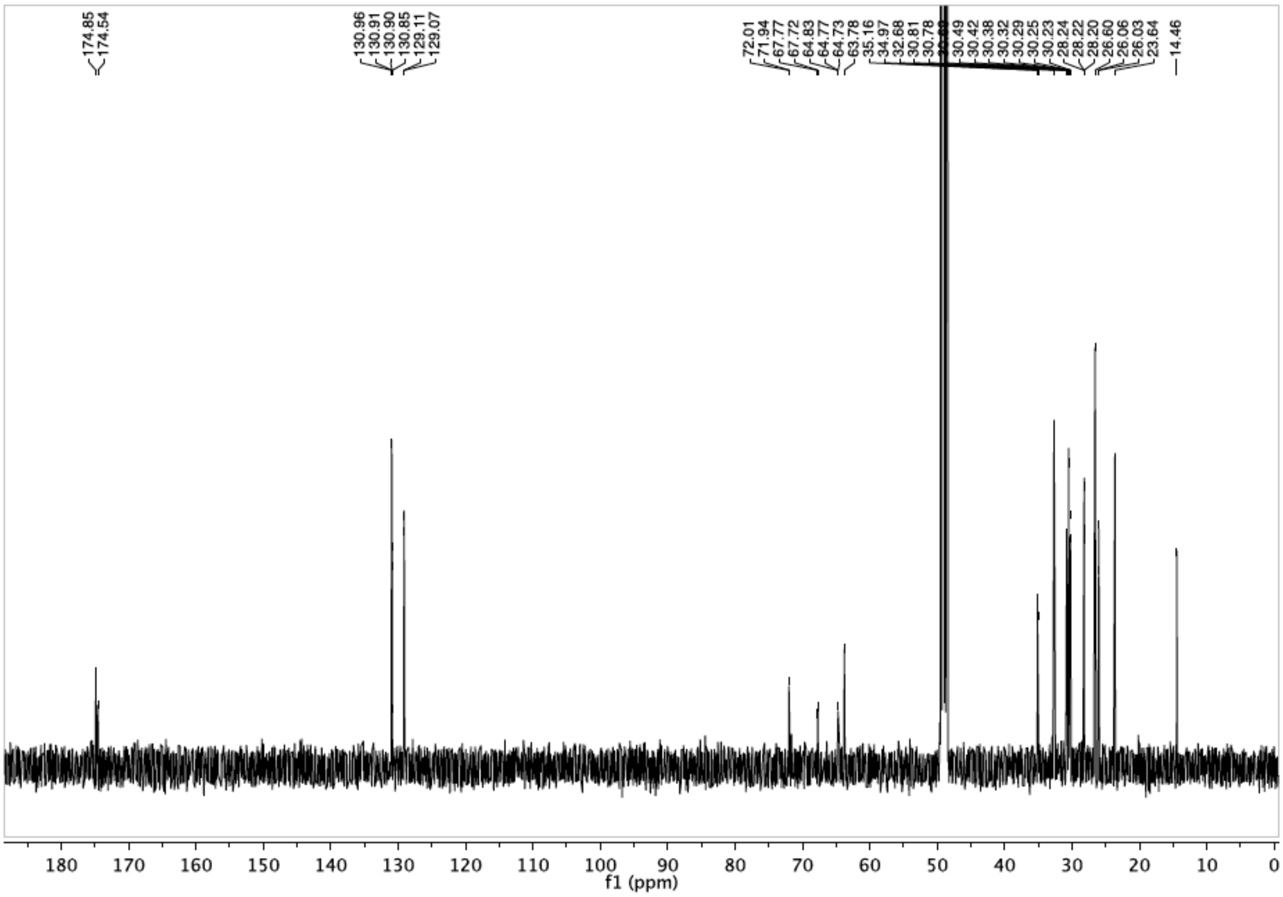

Figure S2. ^{13}C NMR spectrum of natural bovine hearth cardiolipin in CD_3OD

Characterization of the reaction mix containing mono-trans cardiolipin

The reaction mix obtained after 4 min photolysis and work up as previously described was separated into two quantities: one for NMR spectra and the other one treated by GC transesterification for the identification of the cis and trans isomers of linoleic acid, as previously described.

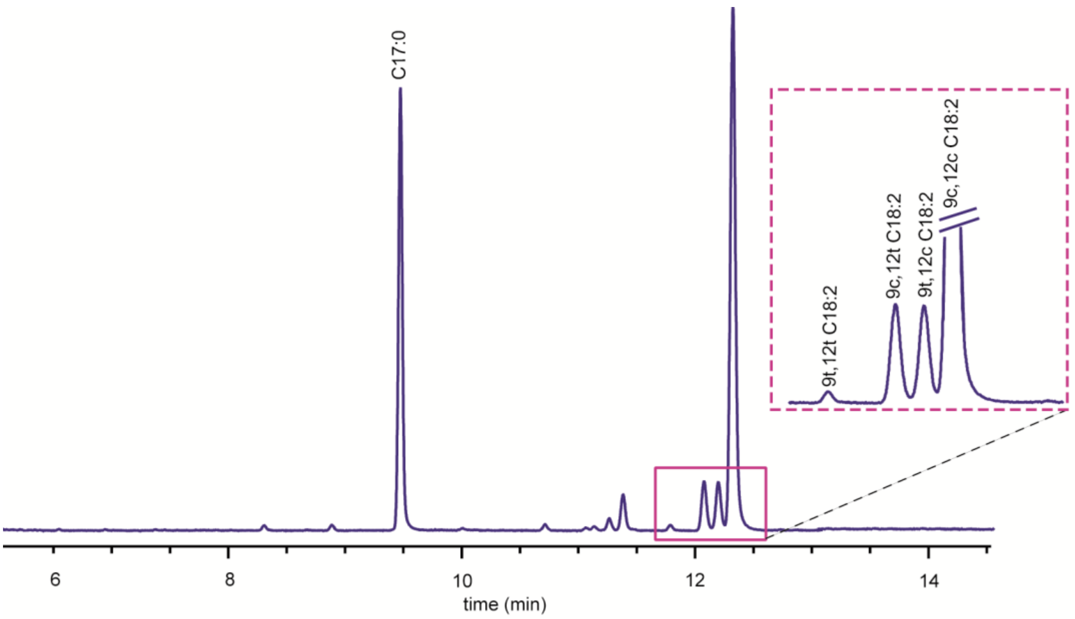

Figure S3 GC analysis of the fatty acid methyl ester (FAME) residues obtained after isomerization of natural bovine hearth cardiolipin in *i*-PrOH for 4 min in the presence of 2-mercaptoethanol and the transesterification reaction following the above reported Experimental conditions. Separation of the four isomers of the linoleic acid residues can be appreciated. In the GC appears also the reference FAME (17:0) used to calibrate and quantitate the transesterification yield. GC peak areas ratio 9c,12c-C18:2/9c,12t-C18:2/9t,12c-C18:2/9c,12c-C18:2 = 84/7.6/7.6/0.8

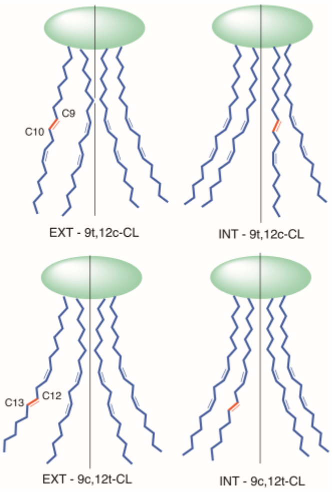

Figure S4. The four possible mono-trans CL structures that are first formed in the step-by-step isomerization process, according to the external or internal fatty acid chain disposition.

^1H NMR (500 MHz, Methanol-d_4) δ 5.35 (tq, *J* = 10.9, 5.5, 3.9 Hz, 15H, C*H*=C*H*), 5.24 (dt, *J* = 8.4, 4.2 Hz, 2H, glycerol CH), 4.55 (bs, 1H, OH), 4.46 (dd, *J* = 12.0, 3.1 Hz, 2H, glycerol CH_2), 4.20 (dd, *J* = 12.0, 6.7 Hz, 2H, glycerol CH_2), 4.01 (t, *J* = 5.5 Hz, 5H, glycerol CH_2), 3.91 (m, 6H, glycerol CH_2), 2.78 (t, *J* = 6.5 Hz, 6H, CH=CHCH_2CH=CH), 2.72 (t, *J* = 6.1 Hz, 1H, CH=CHCH_2CH=CH *trans*), 2.33 (dt, *J* = 14.1, 7.4 Hz, 9H, CH_2CH_2C(O)OR), 2.07 (q, *J* = 6.7 Hz, 15H, CH_2CH=CHCH$_2$CH=CHCH_2), 2.02 – 1.97 (m, 1H, CH_2CH=CHCH$_2$CH=CHCH_2 *trans*), 1.66 – 1.57 (m, 9H, CH_2CH$_2$C(O)OR), 1.41 – 1.26 (m, 67H, CH_2), 0.95 – 0.86 (m, 12H, CH_3) ppm.

^{13}C NMR (126 MHz, Methanol-d_4) δ 174.85, 174.55, 131.70, 131.63, 131.62, 131.34, 131.27, 130.96, 130.89, 130.88, 130.86, 130.83, 129.68, 129.60, 129.12, 129.06, 128.85, 128.78, 71.99, 71.92, 71.58, 71.52, 71.46, 67.75, 67.70, 64.77, 64.72, 63.79, 35.16, 34.97, 33.62, 33.59, 32.95, 32.68, 32.64, 32.53, 31.40, 31.38, 30.81, 30.78, 30.69, 30.49, 30.42, 30.38, 30.32, 30.29, 30.25, 30.23, 30.15, 30.11, 30.07, 28.24, 28.22, 28.21, 28.09, 28.08, 28.05, 26.60, 26.06, 26.03, 23.64, 14.47 ppm.

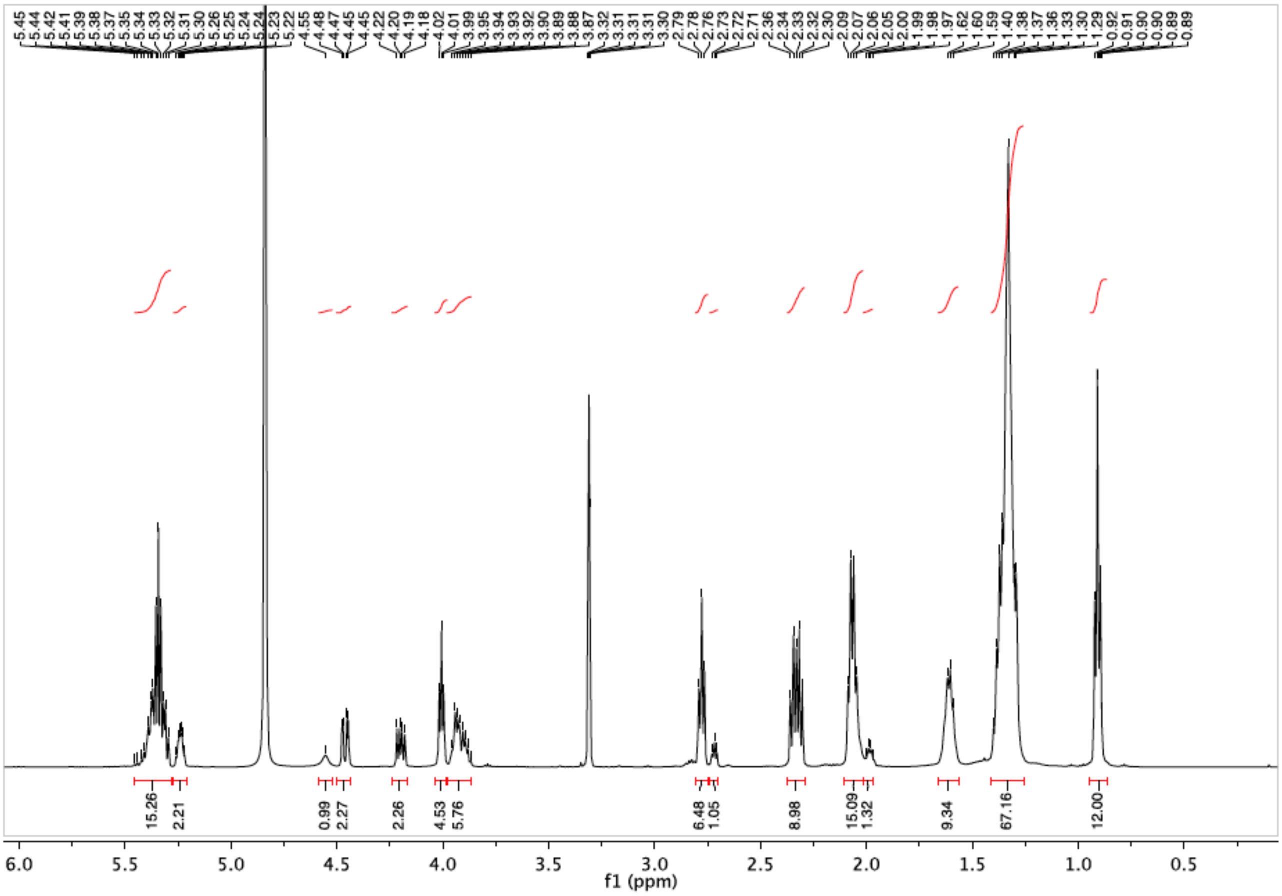

Figure S5 ^1H NMR spectrum in CD_3OD of the reaction mix of CL isomerization after 4 min photolysis under the condition described in the Methods

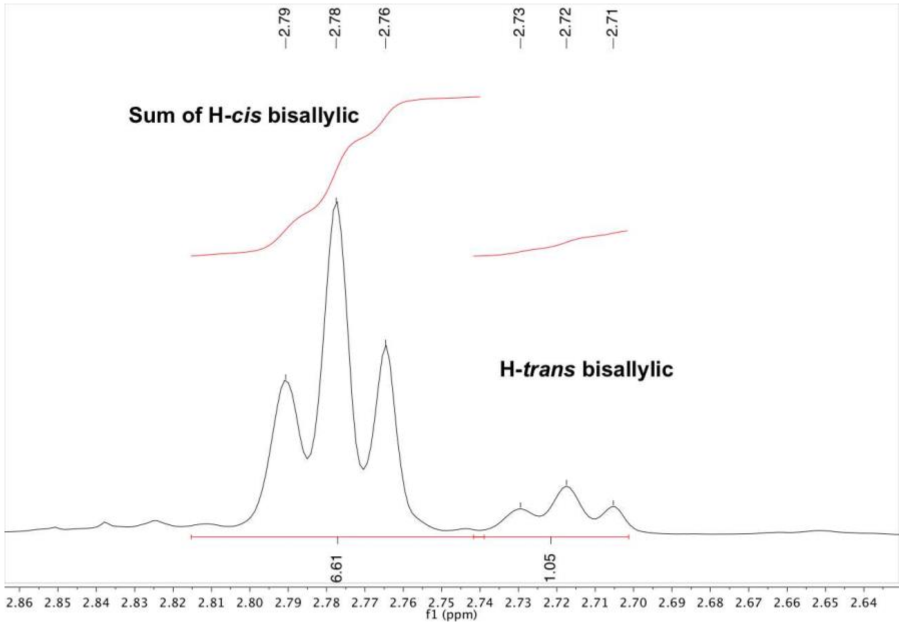

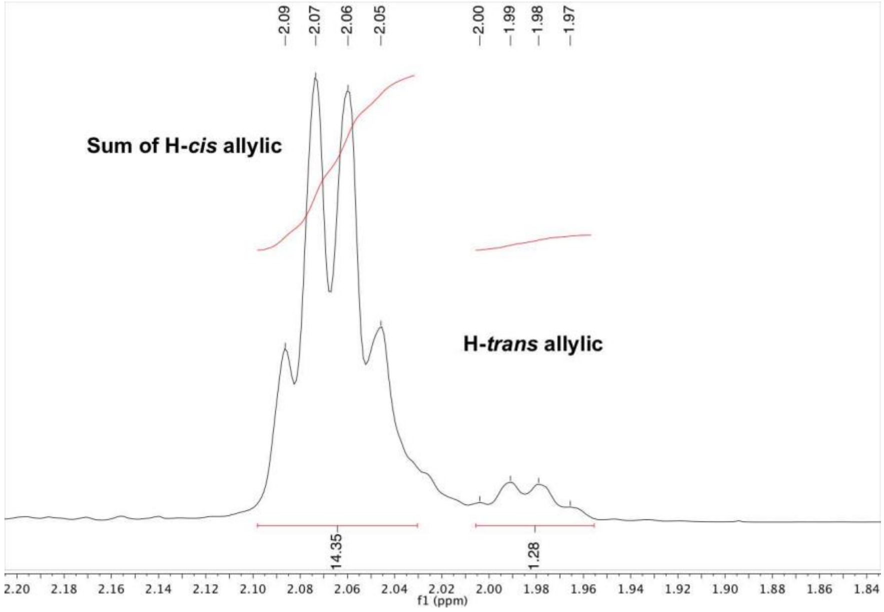

Figure S6 Enlargement of two regions of the ^{1}H NMR spectrum shown in Figure S3. Up: the bis-allylic signals in CL (triplet at 2.78 ppm) and mono-trans CL (triplet at 2.72 ppm) together with their integration ratio (6.6:1). Down: the allylic signals in CL (quadruplet at 2.07ppm) and mono-trans CL (quadruplet at 1.99 ppm) together with their integration ratio (14.3:1.3). Calculating ca 6:1 ratio of the two splitted signals in the mix, it gives ca 15% of mono-trans linoleoyl chains in the mix. Confirmation of such mono-trans isomer percentage comes from the GC analysis under known conditions as described in the Methods.

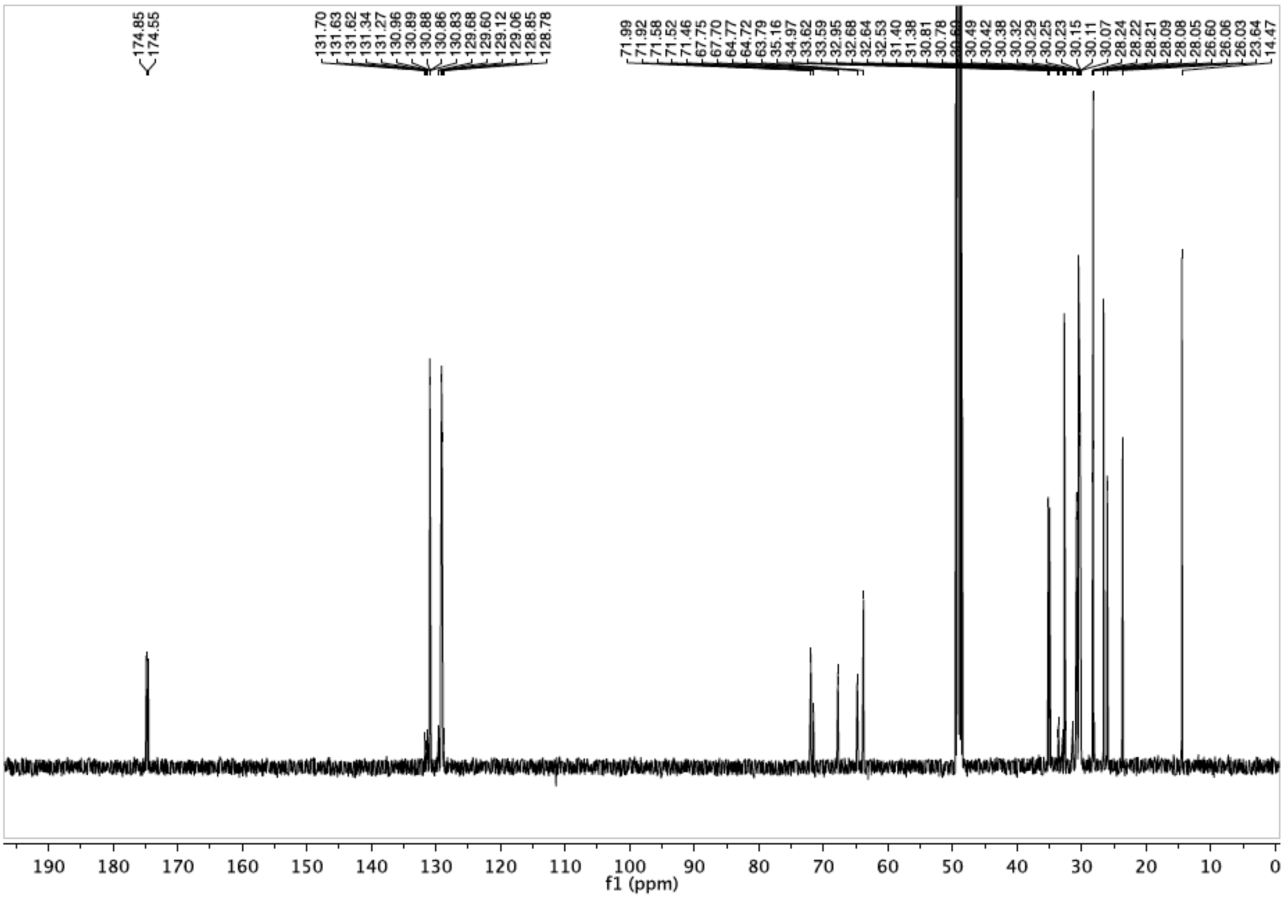

Figure S7 ^{13}C NMR spectrum in CD_3OD of the reaction mix of CL isomerization after 4 min photolysis under the condition described in the Methods.

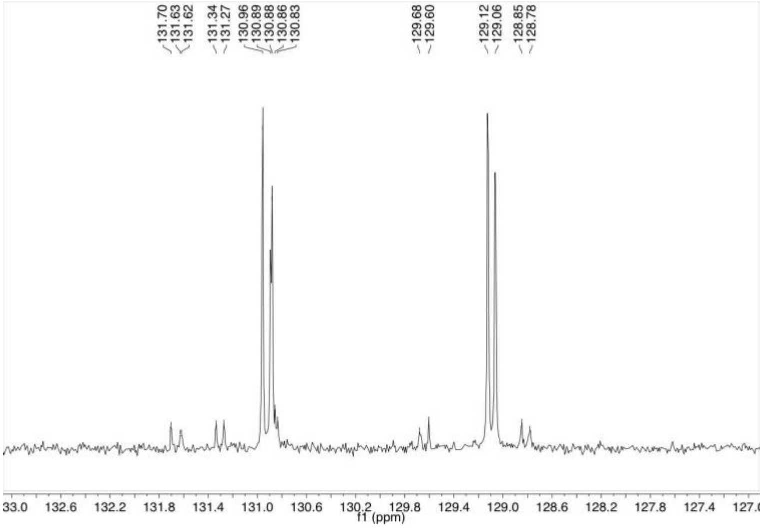

Figure S8. Enlargement of the ^{13}C NMR spectrum in CD_3OD of the reaction mix of CL isomerization evidencing the presence of eight new signals corresponding to the four alkenyl carbon atoms of the main mono-trans isomers of CL formed in the reaction. For discussion and attribution of these peaks see main text.

As detailed in the main text: For 9*cis*,12*trans*-18:2 isomer the literature reported a 0.7 ppm more deshielded resonance,[1] therefore it is possible to attribute the peak at 131.7 ppm (see Figure S6 for details) to the C-13 of a 9*cis*,12*trans*-18:2 CL isomer. By analogy with the CL structure, the signal at 131.6 ppm can be attributed to C-9 in the same molecule. Probably the alkenyl resonances of the other three fatty acid chains of this mono-trans CL remain in the same positions than in the natural cis CL. Examining the resonances at 131.3 and 131.2 ppm again it is possible to attribute to C-9 and C-13 of a second mono-trans CL isomer, by analogy with the 9*trans*,12*cis*-18:2 data.[1] For the signals at 129.7 and 129.6, 128.8 and 128.7 ppm, the attribution of the less deshielded peak at 128.7 ppm is the C-10 of the 9*trans*,12*cis*-18:2 accompanied by the C-12 at 128.8 ppm in the same chain, whereas the resonances at 129.7 and 129.6 ppm individuate the 9*cis*,12*trans*-18:2 CL isomer.

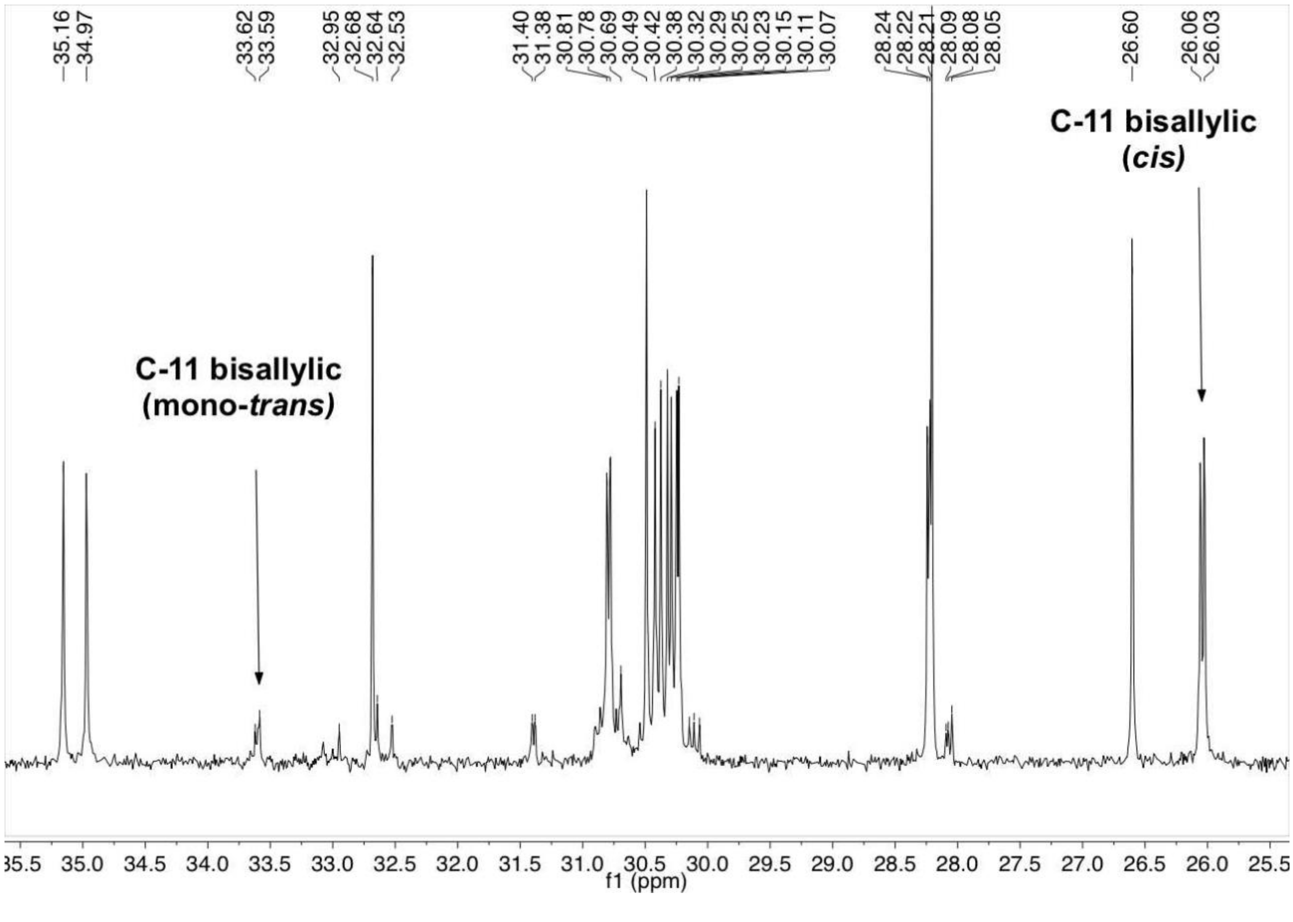

Figure S9. Enlargement of the ^{13}C NMR spectrum in CD_3OD evidencing the region of the alkyl carbon atoms with the presence of the C-11 (bisallylic carbon atom) of the cis and mono-trans linoleic acid structures, which presents a consistent shift of the resonance going from cis to trans geometrical isomer, as described in the literature[5].

The reaction mix obtained after 20 min photolysis and work up as previously described was separated into two quantities: one for NMR spectra and the other one treated by GC transesterification for the identification of the cis and trans isomers of linoleic acid, as previously described.

^{1}H NMR (500 MHz, Methanol-*d*$_4$) δ 5.39 (qq, *J* = 8.7, 5.6, 4.1 Hz, 10H, C*H*=C*H*), 5.28 – 5.21 (m, 2H, glycerol CH), 4.46 (dd, *J* = 12.0, 3.0 Hz, 2H, glycerol CH_2), 4.20 (dd, *J* = 12.0, 6.7 Hz, 2H, glycerol CH_2), 4.01 (t, *J* = 5.4 Hz, 4H, glycerol CH_2), 3.98 – 3.86 (m, 4H, glycerol CH_2), 2.72 (t, *J* = 5.9 Hz, 2H, CH=CHC*H*$_2$CH=CH), 2.65 (s, 2H, CH=CHC*H*$_2$CH=CH *trans*), 2.33 (dt, *J* = 14.3, 7.4 Hz, 8H, CH_2C*H*$_2$C(O)OR), 2.06 (dt, *J* = 11.4, 5.8 Hz, 1H, C*H*$_2$CH=CHCH$_2$CH=CHC*H*$_2$), 2.03 – 1.94 (m, 6H, C*H*$_2$CH=CHCH$_2$CH=CHC*H*$_2$ *trans*), 1.67 – 1.55 (m, 8H, C*H*$_2$CH$_2$C(O)OR), 1.32 (t, *J* = 17.3 Hz, 60H, CH_2), 0.90 (t, *J* = 6.9 Hz, 12H, CH_3).
^{13}C NMR (126 MHz, Methanol-*d*$_4$) δ 174.87, 174.56, 132.00, 131.94, 131.70, 131.64, 131.51, 131.34, 131.29, 129.97, 129.90, 129.67, 129.61, 129.11, 129.07, 128.84, 128.79, 71.97, 71.90, 71.50, 67.75, 67.70, 64.78, 64.73, 63.76, 36.63, 35.15, 35.12, 34.96, 33.64, 33.62, 33.60, 32.65, 32.54, 31.41, 31.39, 30.84, 30.81, 30.80, 30.78, 30.76, 30.74, 30.73, 30.71, 30.50, 30.49, 30.45, 30.43, 30.38, 30.34, 30.24, 30.22, 30.16, 30.13, 28.10, 26.06, 26.03, 23.75, 23.66, 23.64, 23.62, 23.61, 14.47.

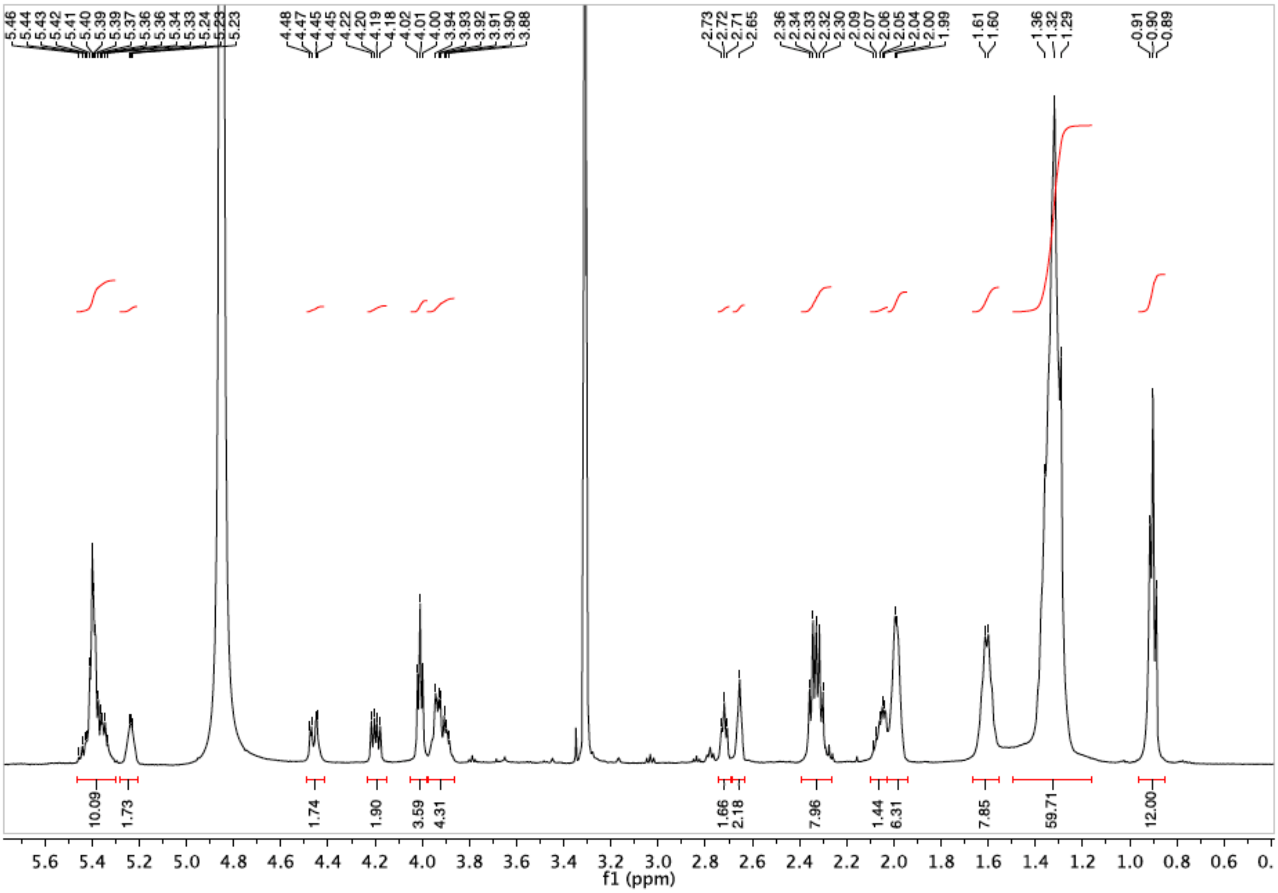

Figure S10 ^{1}H NMR spectrum in CD_3OD of the reaction mix of CL isomerization after 20 min photolysis under the condition described in the Methods.

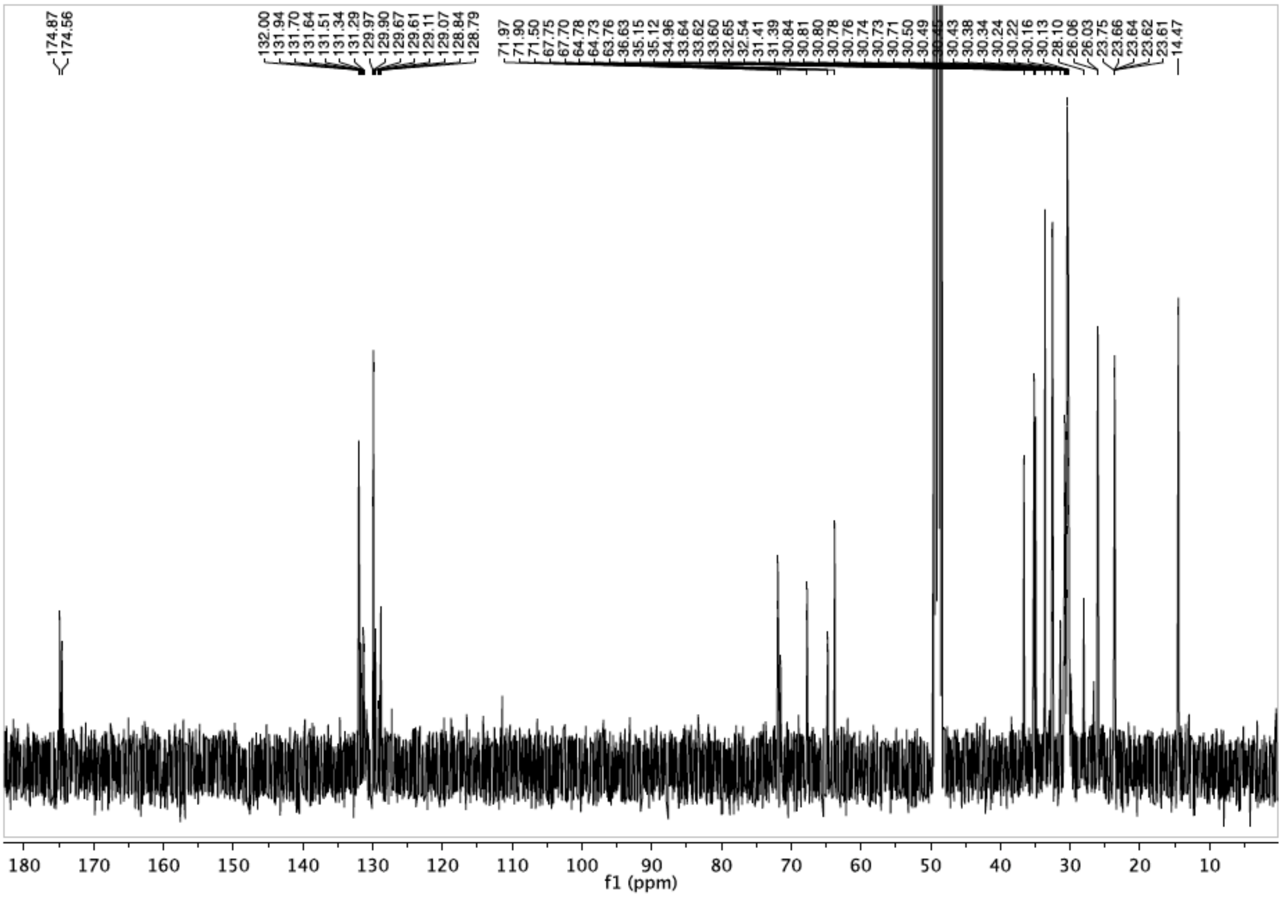

Figure S11 ^{13}C NMR spectrum in CD_4OD of the reaction mix of CL isomerization after 4 min photolysis under the condition described in the Methods.

Ag-complex of lipids and ^{1}H NMR spectra

The use of silver-TLC is well known to separate cis and trans alkenes and was used to separate cis and mono-trans fatty acid isomers in previous works.[2] After preparative Ag-TLC chromatography of the reaction mixture of CL isomerization carried out for 4 min under photolysis conditions, the separation of the Ag-monotransCL complex was achieved. The comparison of the ^{1}H NMR spectra is provided here below.

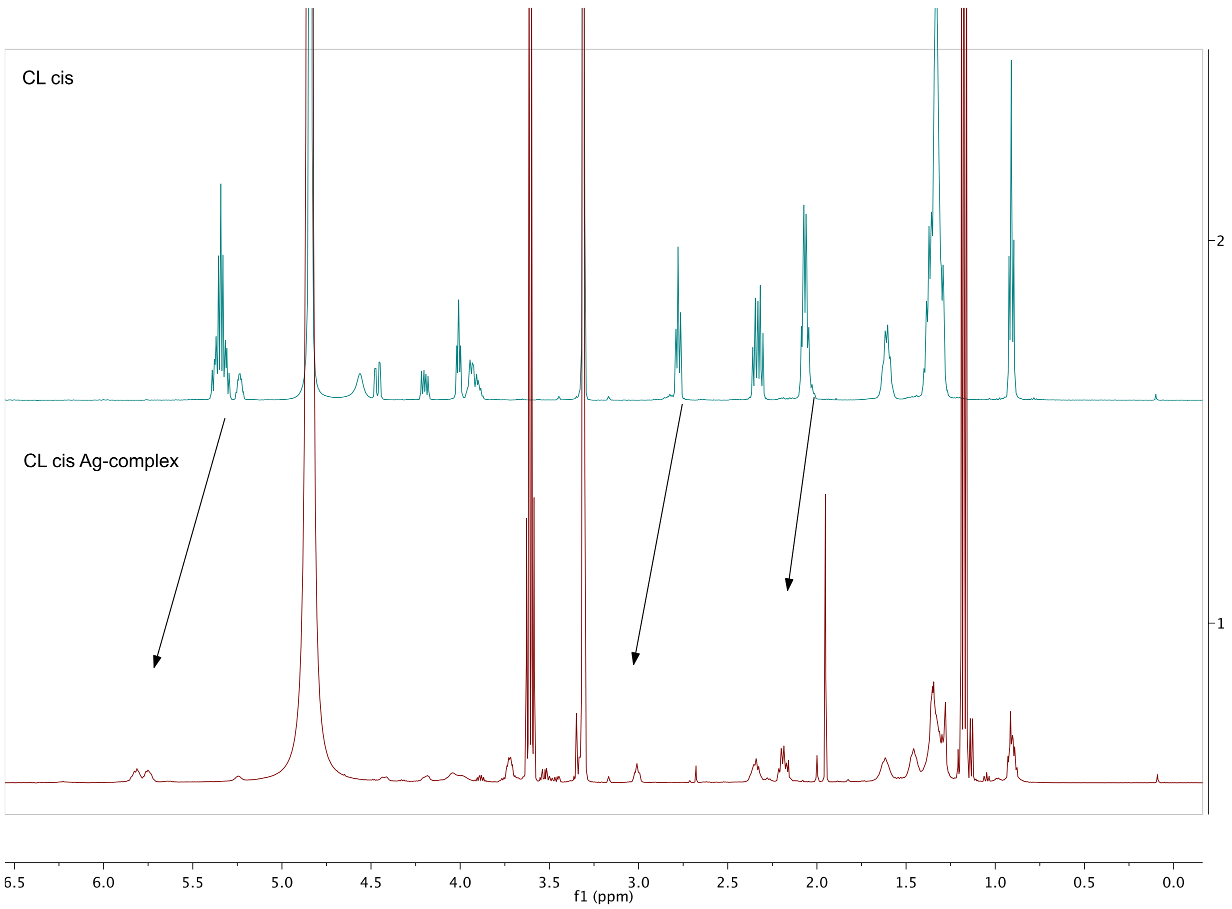

Figure S12 ^{1}H NMR spectra in CD_3OD of CL (up) and Ag-CL complex (down) obtained as described in the Methods.

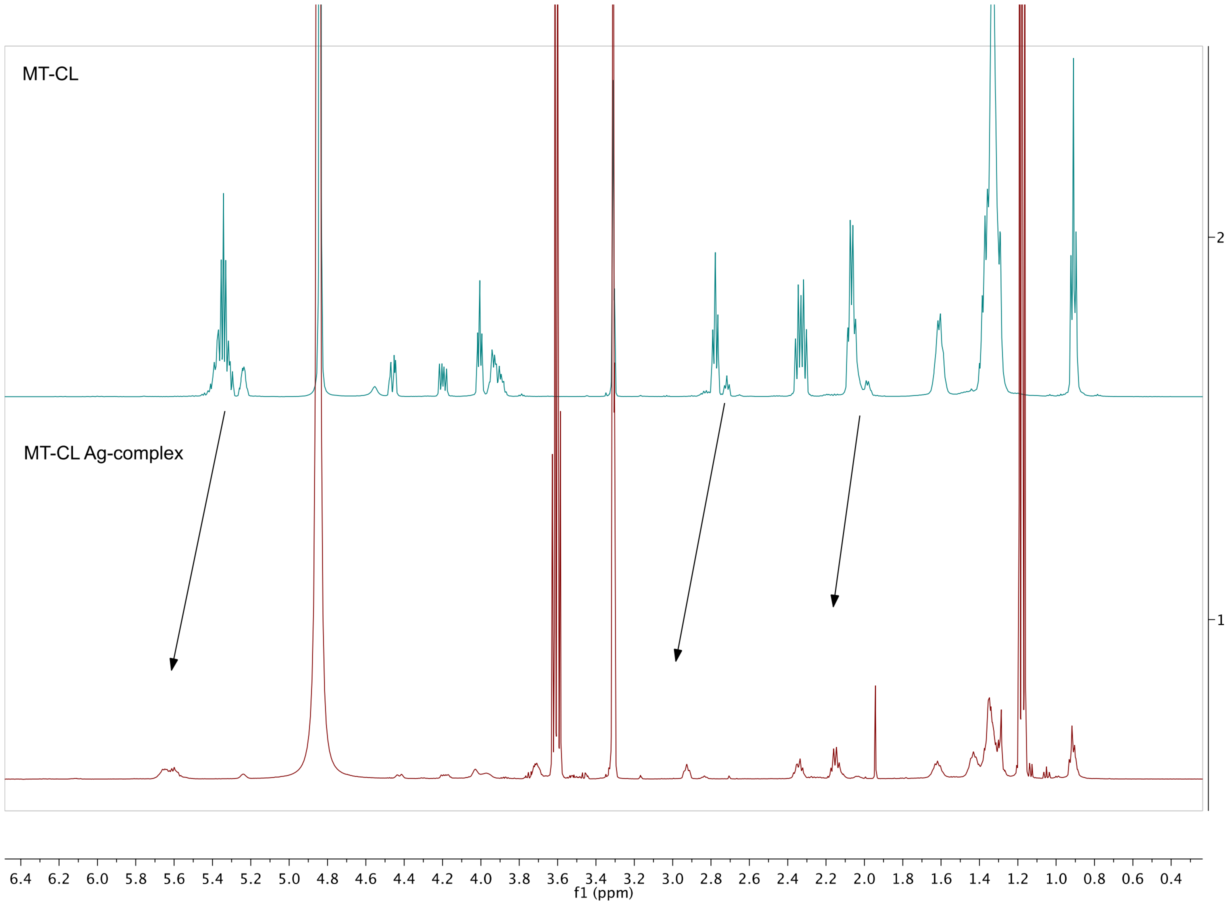

Figure S13 ^{1}H NMR spectra in CD_3OD of monotrans-CL (up) and mono-trans Ag-CL complex (down) obtained as described in the Methods.

Gamma radiolysis experiments

<u>Gamma-radiolysis of CL in *i*-PrOH</u>

A B

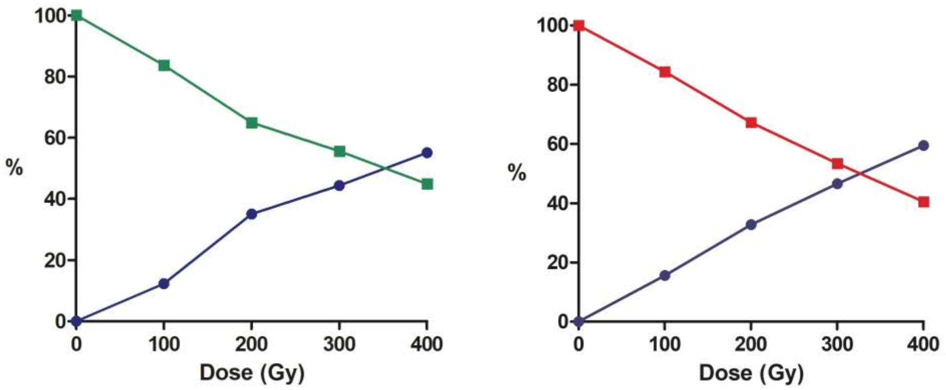

Figure S14. Relative abundance (%) of fatty acid moieties as function of the irradiation dose from the γ-radiolysis of bovine hearth CL (0.35 mM) in isopropanol, in the presence of $HO(CH_2)_2SH$ (0.5 equiv.) and oleic acid methyl ester (9*cis*-18:1, 0.33 equiv.) as internal standard: (**A**) Isomerization of vaccenic acid residues from CL (11*cis*-18:1 green, 11*trans*-18:1 blue) and (**B**) Isomerization of oleic acid methyl ester as internal control (9*cis*18:1 red, 9*trans*-18:1 blue).

A B

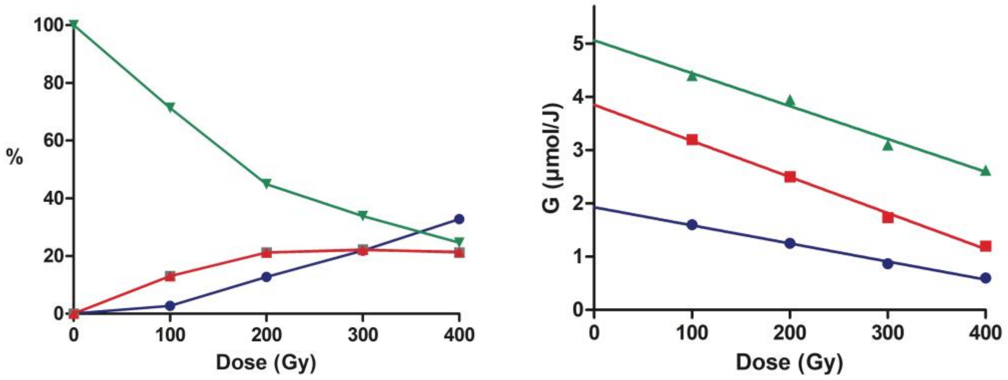

Figure S15. γ-Radiolysis of bovine hearth CL (0.35 mM) in isopropanol, in the presence of $HO(CH_2)_2SH$ (0.5 equiv.) and oleic acid methyl ester (9*cis*-18:1, 0.33 equiv.) as internal standard: (**A**) Relative abundance (%) of L_{ZZ} (green ▼), overlapping L_{ZE} (grey ■) and L_{EZ} (red), and L_{EE} (blue ●) as function of the irradiation dose; (**B**) The chemical radiation yields (*G*) of the disappearance of *G* (–L_{ZZ}) (green ▲) and formation of *G* (L_{EZ} + L_{ZE}) (red ■), *G* (L_{EE}) (blue ●).

Gamma-radiolysis of POPC/CL (75:25) liposomes 0.5 mM in phosphate-buffered water

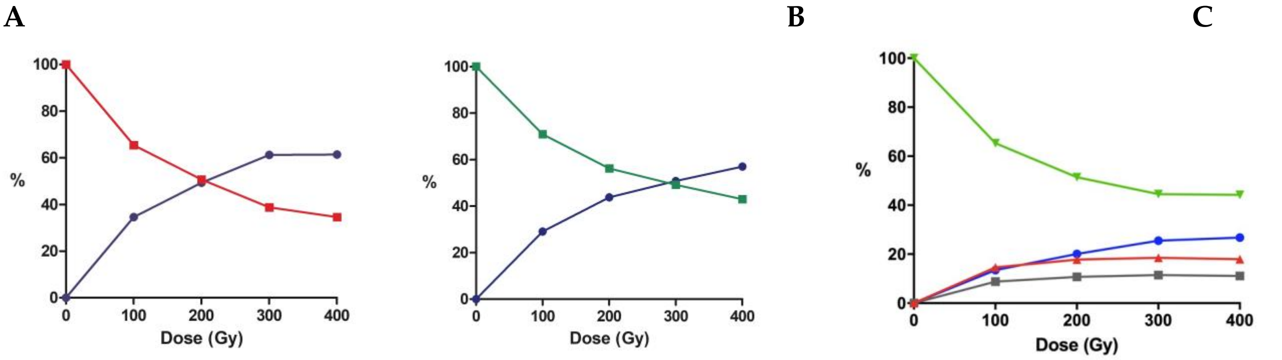

Figure S16. Relative abundance (%) of fatty acid moieties as function of the irradiation dose from the γ-radiolysis of 0.5 mM POPC/CL liposomes (3:1) in the presence of 0.5mM $HO(CH_2)_2SH$: (**A**) Isomerization of oleic acid moieties from POPC (9*cis*-18:1 red, 9*trans*-18:1 blue) (**B**) Isomerization of vaccenic acid moieties from CL (11*cis*-18:1 green, 11*trans*-18:1 blue); (**C**) Isomerization of linoleic acid moieties from CL (L_{ZZ} (green ▼), L_{ZE} (grey ■), L_{EZ} (red ▲), and L_{EE} (blue ●).

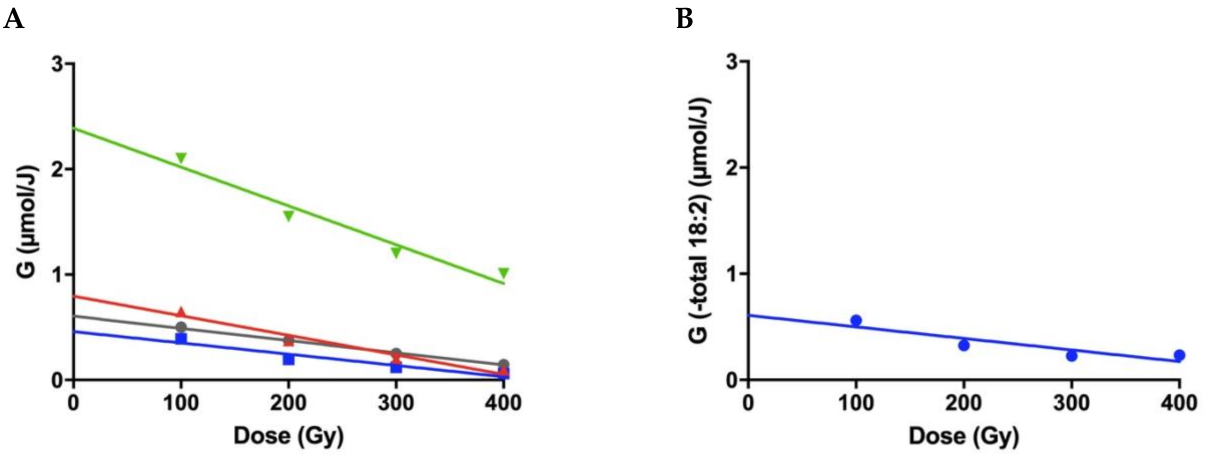

Figure S17. γ-radiolysis of 0.5 mM POPC/CL liposomes (3:1) in the presence of 0.5mM $HO(CH_2)_2SH$; (**A**) The chemical radiation yields (*G*) of the disappearance of $L_{Z,Z}$ (green ▼) and formation of L_{ZE} (grey ●), L_{EZ} (red ▲), L_{EE} (blue ■) versus dose; (**B**) the G for the consumption of all geometrical isomers (total L) as a function of the irradiation dose.

(1) (5) Bus, J.; Sies, I.; Lie Ken Jie, M. S. F., 13C-NMR of methyl, methylene and carbonyl carbon atoms of methyl alkenoates and alkynoates, *Chem. Phys. Lipids* **1976**, *17*, 501–518.

(2) Menounou, G.; Giacometti, G.; Scanferlato, R.; Dambruoso, P.; Sansone, A.; Tueros, I.; Amézaga, J.; Chatgilialoglu, C.; Ferreri, C. *Chem. Res. Toxicol.* **2018**, *31*, 191–200.
